# Multicentric pheochromocytoma and involvement of the inferior vena cava

**DOI:** 10.1590/S1516-31802001000200010

**Published:** 2001-03-02

**Authors:** Antonio Marmo Lucon, Renato Falci, José Nery Praxedes, Marcel Cerqueira Cesar Machado, Luis Balthazar Saldanha, Marcelo Marcondes Machado, Sami Arap

**Keywords:** Pheochromocytoma, Retroperitoneal neoplasia, Adrenal tumor, Adrenal surgery, Vena cava, Feocromocitoma, Neoplasia retroperitoneal, Tumor adrenal, Veia cava

## Abstract

**CONTEXT:**

Extension of pheochromocytomas to the inferior vena cava is rare. Multicentric tumors are rare as well, being present in up to 10% of cases. Surgery is the treatment of choice because of the longterm survival free of disease.

**DESIGN:**

Case report.

**CASE REPORT:**

We report on a case of right adrenal pheochromocytoma with extension to the supradiaphragmatic vena cava, which underwent surgical excision through thoracophrenic laparotomy without the need for cardiopulmonary bypass. In a 6-year follow-up, another pheochromocytoma was found in the infra-renal Zuckerkandl's organ. Complete surgical excision of the tumor was performed by a median laparotomy and complete retroperitoneal dissection. In both cases, the total removal of the pheochromocytoma has been guaranteed by having margins free of tumor and a normal postoperative level of catecholamines. The pathological study revealed a malignant pheochromocytoma with margins free of neoplasia in both specimens.

## RESUMO

**CONTEXTO:**

Feocromocitoma com invasão de veia cava inferior é raro. Tumores multicêntricos são igualmente raros, estando presentes em até 10% dos casos. A cirurgia é o tratamento de escolha, uma vez que a sobrevida livre da doença é longa.

**TIPO DE ESTUDO: Relato de caso.:**

RELATO DE CASO: Relatamos um caso de feocromocitoma de adrenal direita com extensão para veia cava inferior supra-diafragmática, retirado cirúrgicamente através de tóraco-freno-laparotomia, sem a necessidade de circulação extra-corpórea. Após seis anos de seguimento, outro feocromocitoma foi achado no órgão de Zuckerkandl. A excisão cirúrgica completa do tumor foi realizada através de laparotomia mediana e dissecção retroperitoneal. Em ambos os casos, margens cirúrgicas livres do tumor e níveis pós-operatórios normais de catecolaminas garantiram remoção total do feocromocitoma. O estudo anatomopatológico revelou feocromocitoma maligno com margens livres de neoplasia em ambos os espécimes.

## INTRODUCTION

Pheochromocytomas are tumors that arise in the adrenal medulla or in other foci of chromaffin cells and most of them are benign. Some 10% of pheochromocytomas are extra-adrenal, the majority of them being found throughout the length of the sympathetic cell chain, which contains chromaffin cells, whether in the head, the neck, the thorax or the abdomen. The most common site of extra-adrenal pheochromocytoma is Zuckerkandl's organ.^1^ The excessive secretion of catecholamines by the tumor is responsible for most of the adrenergic signs and symptoms such as hypertension, headache and tachycardia, among others.

Although pheochromocytomas are rarely a cause of hypertension (less than 1% of the hypertensive population), their diagnosis is important by virtue of the potential cure of the hypertension which it represents, and also because of the oncological character of the lesion.^2^ The available literature reports only 16 cases of this tumor with invasion of the inferior vena cava.^3-18^ When two or more tumors are found in a patient, the possibility of a family disease as well as multiple endocrine neoplasia should be investigated. The family pheochromocytoma may also be associated with Von Hippel Lindau and Von Recklinghausen's disease.^19^

Our purpose is to report a case of malignant right adrenal pheochromocytoma with vena cava invasion and its surgical approach.

## CASE REPORT

A 43-year-old man was admitted for investigation of recent hypertension onset associated with headache and tremors. Urinary vanillimandelic acid (VMA) was 16 mg/24h (normal range 2 to 12 mg/24h) and the urinary methanephrines were 2.1 mcg/mg of creatinine (normal ranges 0.05 to 1.2). Abdominal ultrasonography showed a solid lesion measuring 6.0 × 6.5 cm in the topography of the right adrenal. A computerized tomography (CT) scan confirmed the ultrasonography findings and revealed its extension into the inferior vena cava above the diaphragm without reaching the right atrium.

**Figure 1:**
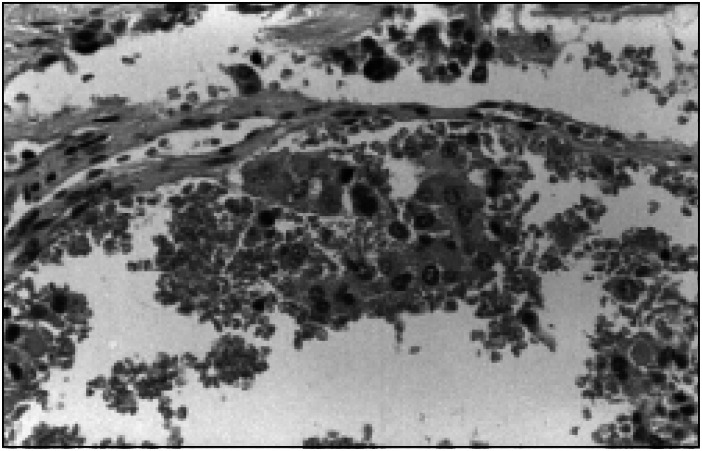
Hematoxylin-eosin staining showing vascular invasion of the malignant pheochromocytoma.

A right thoracophrenic laparotomy allowed good access to the tumor and inferior vena cava. First, the supra-diaphragmatic inferior vena cava was dissected and repaired. The same procedure was used to control the left renal vein and inferior vena cava below the renal vein. The tumor and the right kidney were isolated and removal en bloc was performed, preserving the right adrenal vein with its tumoral thrombi inside. Vascular clamps were then placed on the infra-renal vena cava, left renal vein, hepatic pedicle (Pringle's maneuver) and intra-thoracic inferior vena cava. The anterior wall of the abdominal vena cava was opened and the thrombi were removed using Randall's clamps. Flushing with 1:200 heparin solution was carried out. At this time, the bleeding from the lumbar veins was not serious and did not jeopardize the removal of the thrombi with good visibility. The vessels were closed with a running prolene suture. During the period of clamping, which lasted for 12 minutes, there was light arterial hypotension, controlled by the anesthesiologist. The clamps were removed in reverse order (Figure 1).

**Figure 2:**
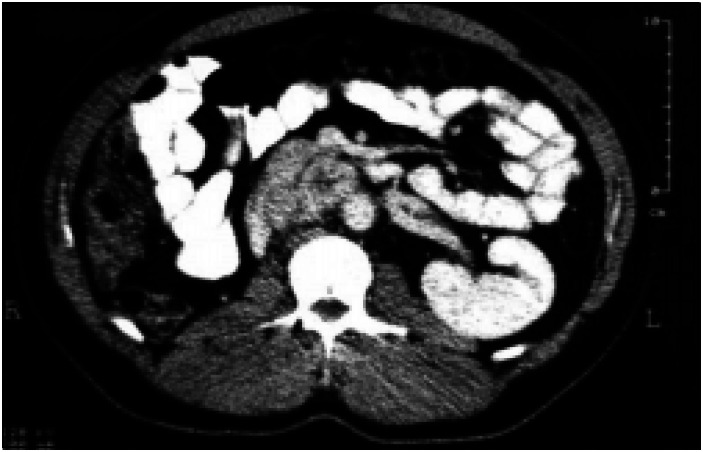
CT scan of the abdomen showing a 3 × 4 cm pre-aortic mass.

The patient became free of symptoms for six years, with normal catecholamine serum levels. After this period, a rise in arterial blood pressure level was found during a medical follow-up assessment. The norepinephrine serum level was found to be 3347 (normal 40 to 268 pg/ml) and urinary norepinephrine 741 (normal 65 to 400 mcg/24h). The epinephrine serum level was normal. The CT scan of the abdomen revealed a pre-aortic mass of 3 × 4 cm situated immediately below the renal artery (Figure 2). The existence of the mass was confirmed by magnetic resonance imaging (MRI), appearing with hypersignal in T2. The radioisotope study with meta-iodobenzyl-guanidine (MIBG) demonstrated considerable enhancement of the tumor.

The surgical removal of the mass described was undertaken by median laparotomy with retroperitoneal dissection. A frozen biopsy of the specimen showed free margins. The normal postoperative catecholamine levels confirmed the total removal of the tumor.

Alpha and beta-blockers (prazosin and propranolol, respectively) were used as preoperative treatment in both operations. The adrenergic blockage was begun 15 days before the surgery with gradual adjustment of dosage, reaching 6 mg of prazosin and 80 mg of propranolol per day. The pathological study of the specimen showed malign pheochromocytoma with free margins. The patient is well after a 7-year follow-up.

## DISCUSSION

The invasion of the inferior vena cava has already been described in relation to many abdominal tumors. The retroperitoneal tumor most commonly associated with extension into this vessel is renal cell carcinoma^12^ but it rarely occurs in pheochromocytomas. The extension may occur by direct invasion of the vessel wall or by luminal progression within the vein. The direct invasion is more frequently observed in malignant neoplasia.^17^ The technique of choice employed by many authors for the removal of tumors that present a thrombus inside the infra or supra-hepatic vena cava without affecting the right atrium utilizes extra corporeal circulation with hypothermia and total cardiac arrest for their safe removal.^12^ However, in such cases, radical surgery is also practicable without the use of extra corporeal circulation,^20^ thus avoiding the morbidity related to this procedure as well as the increase in hospital costs, as demonstrated in our case.

Others prefer to use the piggyback style of mobilization of the liver to access the inferior vena cava, which is the technique employed for orthotopic liver transplantation.^21^ The extension of the tumor into the inferior vena cava with no invasion of the vessel wall does not necessarily mean malignancy,^22^ which occurs when there is direct invasion of the vessel wall.^23^ The presence of distant metastases is the unquestionable criterion for the classification of malignant pheochromocytoma.^24^ Although malignant pheochromocytomas tend to reappear or present metastases during long-term follow-up, surgical removal of these tumors is the best option for treatment,^25^ considering that the patients have a ten year survival rate with no evidence of disease.

The incidence of extra adrenal pheochromocytomas or multicentric tumors varies from 3.8 to 10%,^26,27^ the most common site being Zuckerkandl's organ. In these cases, where the concentration of norepinephrine is considerably greater than that of epinephrine, the existence of extra adrenal pheochromocytoma may be suspected, as the methylation occurs mainly in the adrenal gland. This was observed in the case presented. In rare cases, measurement of plasma catecholamine values in blood samples taken from different levels of the inferior vena cava may also be useful in the search for possible sites of metastases.^26^

At present, MRI is tending to become the method of choice for the assessment of thrombi in the large vessels, visualized mainly in T1 and T2 sequences.^17^ In the past, laparotomy was the approach of choice for pheochromocytomas because of the frequency of bilateral disease and the possibility of the existence of extra adrenal tumors misdiagnosed prior to surgery. Nevertheless, with the improvement in methods of localization such as CT scan, MRI and MIBG scintigraphy, the surgeon enjoys the possibility of choosing the best surgical approach beforehand.

The use of alpha and beta blocking agents preoperatively ensures a fully expanded vascular system and also minimizes intraoperative blood pressure fluctuation. Recently, the use of other drugs such as calcium channel blockers, instead of alpha and beta adrenergic antagonists has been demonstrated.^28^ However, due to the effectiveness and low costs of alpha and beta blocking agents, they are still being used as drugs of choice in our experience.
